# Quality of life and fatigue of patients with spinal bone metastases under combined treatment with resistance training and radiation therapy- a randomized pilot trial

**DOI:** 10.1186/1748-717X-9-151

**Published:** 2014-07-07

**Authors:** Harald Rief, Michael Akbar, Monika Keller, Georg Omlor, Thomas Welzel, Thomas Bruckner, Stefan Rieken, Matthias F Häfner, Ingmar Schlampp, Alexandros Gioules, Jürgen Debus

**Affiliations:** 1 Department of Radiation Oncology, University Hospital of Heidelberg, Im Neuenheimer Feld 400, Heidelberg 69120, Germany; 2 Department of Medical Biometry, University Hospital of Heidelberg, Im Neuenheimer Feld 305, Heidelberg 69120, Germany; 3 Department of Orthopaedics and Trauma Surgery, University Hospital of Heidelberg, Schlierbacherstrasse 120a, Heidelberg 69118, Germany; 4 Department of Psychooncology, University Hospital of Heidelberg, Im Neuenheimer Feld 400, Heidelberg 69120, Germany

## Abstract

**Background:**

The aim of this trial was to compare the effects of resistance training versus passive physical therapy on quality of life (QoL), fatigue, and emotional distress outcomes during radiation therapy in patients with spinal bone metastases under radiotherapy (RT).

**Methods:**

In this randomized trial, 60 patients were treated from September 2011 until March 2013 into one of the two groups: isometric resistance training or physical therapy with thirty patients in each group during RT. EORTC QLQ-BM22, EORTC QLQ-FA13, and FBK-R10 were assessed at baseline, three months, and six months after RT.

**Results:**

Psychosocial aspects in resistance training group (Arm A) were significantly improved after three (p = 0.001) and six months (p = 0.010). Other rated items of the QLQ-BM22 painful site, and pain characteristics were without significant differences. Functional interference showed a positive trend after six months (p = 0.081). After six months, physical fatigue (p = 0.013), and interference with daily life (p = 0.006) according to the QLQ-FA13 assessment improved in Arm A significantly. Emotional distress was in Arm A lower after six months (p = 0.016). The Cohen’s effect size confirmed the clinically significant improvement of these findings.

**Conclusions:**

In this group of patients we were able to show that guided isometric resistance training of the paravertebral muscles can improve functional capacity, reduce fatigue and thereby enhance QoL over a 6-months period in patients with stable spinal metastases. The results offer a rationale for future large controlled investigations to confirm these findings.

**Trial registration:**

Clinical trial identifier NCT01409720

## Background

Spinal bone metastases of an advanced stage of a malignant primary disease are frequently associated with a decline of quality of life (QoL) and fatigue. Approximately 70% of cancer patients report experiences of fatigue during chemo- or radiotherapy [1]. The effects of bone metastases result in resting pain and during physical stress, limitation in daily life, lower performance ability, risk of pathological fractures and neurological deficits. Pain is the most significant factor for reduced QoL of patients with bone metastases. Fatigue is one of the most prevalent and distressing symptoms reported by cancer patients [2] and is described as an unusual feeling of exhaustion, tiredness, feebleness, and reduced performance ability [3]. Cancer-related fatigue is a serious problem that impairs patients physically, mentally, and socially [4,5] and previous trials have identified these symptoms in patients with an advanced stage of a malignant primary disease.

Although there has been frequent research throughout recent decades, physical exercise for cancer patients is a potential and effective intervention to treat fatigue and to improve QoL during RT [6-12]. Physical exercise with spinal bone metastases was considered inappropriate due to the risk of pathological fractures and spinal cord compression [13]. Accordingly the effect of resistance training during RT of patients with spinal bone metastases is still unknown. The aim of this randomized trial was to compare the effects of resistance training versus passive physical therapy on quality of life, fatigue, and emotional distress outcomes during radiation therapy in patients with spinal bone metastases.

## Methods

### Subjects and recruitment

**Figure 1:**
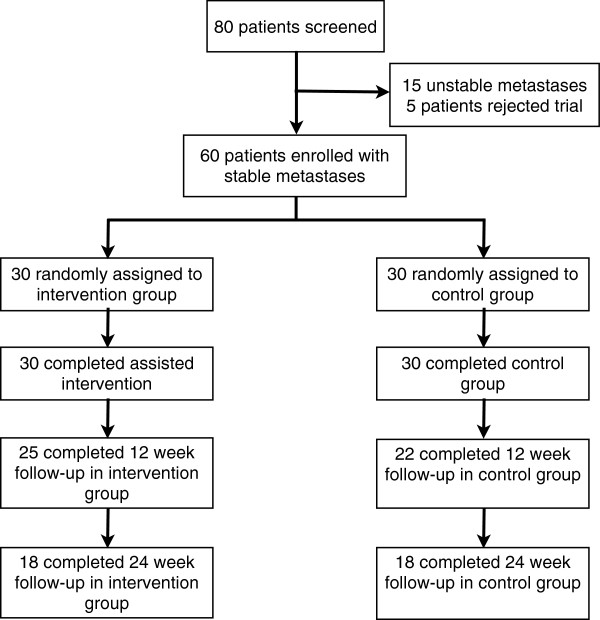
Flow of participants through the trial.

From September 2011 through March 2013, consecutive 80 patients with a histologically confirmed cancer of any primary and bone metastases of the thoracic or lumbar segments of the vertebral column, or of the os sacrum were considered in the Radiooncology Department of the Heidelberg University Clinic. Inclusion criteria were an age of 18 to 80 years, a Karnofsky performance score [14] ≥ 70, written consent to participate, and already initiated bisphosphonate therapy. The patients were subjected to a staging of their vertebral column within the context of the CT designed to plan the radiation schedule prior to enrolment into the trial. In this examination metastases in the thoracic and lumbar spine were classified as “stable” or “unstable”. This was diagnosed independently by a specialist for radiology as well as by a specialist for orthopedic surgery. The specifications for an unstable vertebral body were tumor occupancy more than 60% of the vertebral body, and pedicle destruction [15]. Only a metastasis classified by both specialists as “stable” was suggested eligible for inclusion. Out of 80 patients considered eligible 15 patients were excluded due to unstable metastases, and five patients declined to participate in the study. The initial sample size 60 patients fulfilled the inclusion and were enrolled into the trial (Figure 1). The study was approved by the Heidelberg Ethics Committee (Nr. S-316/2011).

### Design, randomized allocation, and procedures

This is a randomized, controlled, explorative intervention trial in the parallel group design with the intention to compare the effects of quality-of-life and fatigue of a resistance training program for strengthening the paravertebral muscles in patients with spinal bone metastases as an adjunct to radiotherapy (RT). Patients in the control group conducted physical therapy in the form of breathing exercises. A block randomization approach was used. After the baseline measurements, the patients with stable bone metastases were assigned to the respective treatment arms on a 1:1 basis according to the randomization list. The randomization procedure was carried out by a central office. Arm A (intervention group, resistance training) and in Arm B (control group, physical therapy) each consisted of 30 patients. The target parameters were measured at the start of radiotherapy (t_0_), after twelve weeks (t_2_), and after six month (t_3_). The target parameters comprise the documentation and completion of the questionnaires EORTC QLQ-BM22, EORTC QLQ-FA13, QSC-R10, and the recording of patient-specific data. The data of the patient records were collected by the authors. Patient characteristics are shown in Table 1.

**Table 1:** Patient characteristics at baseline

		**Intervention group (n = 30)**		**Control group (n = 30)**	
		**n**	**%**	**n**	**%**
Age (years)					
	Mean (SD)	61.3 (10.1)		64.1 (10.9)	
Gender					
	Male	14	46.7	19	63.3
	Female	16	53.3	11	36.7
Body Mass Index					
	Mean (SD)	25.3 (4.5)		24.4 (3.6)	
Karnofsky PS (median, range)		80 (70-100)		80 (70-100)	
Primary site					
	Lung	12	9.2	8	26.6
	Breast	5	16.7	6	20.1
	Prostate	5	16.7	9	30.1
	Melanoma	1	3.3	1	3.3
	Kidney	1	3.3	2	6.7
	Other	6	20.1	4	13.4
Spinal metastases site					
	Thoracic	17	56.7	14	46.7
	Lumbar	9	30.0	13	43.3
	Thoracic and lumbar	2	6.7	2	6.7
	Sacrum	2	6.7	1	3.3
Number of metastases					
	Mean (range)	1.4 (2-4)		1.7 (1-5)	
	Solitary	22	73.3	18	60.0
	Multiple	8	26.7	12	40.0
Type of metastases					
	Mixed	2	6.7	2	6.7
	Osteoblast	9	30.0	10	33.3
	Osteolytic	19	63.3	18	60.0
Concomitant metastases at baseline					
	Visceral	12	40.0	5	16.7
	Brain	3	10.0	3	10.0
	Lung	7	23.3	4	13.3
	Tissue	8	26.7	6	20.0
Hormonotherapy		10	33.3	16	53.3
Immunotherapy		7	23.3	5	16.7
Chemotherapy		25	83.3	20	66.7
Pathological fracture at baseline		6	20.0	9	30.0
Neurological deficit		0	0.0	2	6.7

SD Standard deviation.

### Study interventions

The interventions commenced on the same day as radiotherapy and were performed on each day of RT treatment (Monday through Friday) over a two-week period, independent of the number of fractions. During the two-week RT period, the patients in the resistance training group (Arm A) performed the exercises under the guidance of a physiotherapist. The patients were then instructed to practice the training in their homes three times a week and continued the resistance training themselves until the last investigation after six months. The resistance training lasted approx. 30 min, the physical therapy (Arm B) approx. 15 min. Since the site of the bone metastases differed from patient to patient, three different exercises were enacted to ensure an even isometric resistance training of the muscles along the entire vertebral column. Patients in the control group (Arm B) received passive physical therapy in form of breathing exercises also for a period of two weeks. The passive physical therapy was conducted for patients’ compliance, and avoidance of a high drop out rate. A detailed report of the intervention and its application has already been published [16].

### Measures of primary and secondary End points

The primary endpoint was QoL, assessed using the EORTC QLQ BM22 questionnaire, which is specially designed for patients with bone metastases. The QLQ BM22 module (range 0-100) comprises 22 items and four scales for the measurement of pain in various parts of the body (painful sites), pain characteristics (persistent pain, recurrent pain), functional impairment (occurrence of pain when performing different activities, interference with everyday activities), and psychosocial aspects (family, worries, hope) [17]. Secondary endpoints were fatigue, and emotional distress. Fatigue was assessed using the EORTC QLQ FA13 (range 0-100). This QLQ FA13 module includes 13 items and five scales for measuring cancer-related fatigue [18], with subscales covering physical fatigue, emotional fatigue, cognitive fatigue, interference with daily life, and social sequelae. Emotional distress was assessed using the QSC-R10 (range 0-50) questionnaire. The QSC-R10 [19] module is a valid and reliable questionnaire for determining emotional distress and anxiety in cancer patients [20]. The questionnaires were filled out by the patients at the study site.

### Radiotherapy

Radiotherapy was performed in the Radiooncology Department of the Heidelberg University Clinic. After virtual simulation was performed to plan the radiation schedule, radiotherapy was carried out over a dorsal photon field of the 6MV energy range. PTV covered the specific vertebral body affected as well as the ones immediately above and below. In Arm A 24 patients (80%) were treated with 10 × 3 Gy, three patients (10%) with 14 × 2.5 Gy, and three patients (10%) with 20 × 2 Gy. In Arm B the dose fractions for 28 patients (93.3%) were 10 × 3 Gy, for one patient (3.3%) 14 × 2.5 Gy, and for one patient (3.3%) 20 × 2 Gy. The median individual dose in all patients was 3 Gy (range 2-3 Gy), the median total dose 30 Gy (range 20-35 Gy). The individual and total doses were decided separately for each individual patient, depending on the histology, the patient’s general state of health, and on the current staging and the corresponding prognosis.

### Sample calculation and statistical analysis

The total number of patients undergoing radiotherapy in the radiation oncology department of the Heidelberg University Clinic for metastatic processes in the vertebral column in the recruitment period is approx. 120, about 90 of whom shall fulfill the inclusion criteria. On account of the explorative character of this study it was not possible to estimate the total number of cases; with a scheduled number of 30 patients per group, it will, however, be possible to detect a standardized effect (Cohen’s d) of about 0.8 with a power of 80% and a significance level α of 5%. All variables were analyzed descriptively by tabulation of the measures of the empirical distributions. According to the scale level of the variables, means, standard deviations, medians as well as minimum and maximum or absolute and relative frequencies, respectively, will be reported. Additionally, for variables with longitudinal measurements, the time courses of individual patients and summarized by treatment groups. Descriptive p-values of the corresponding statistical tests comparing the treatment groups will be given. Wilcoxon signed rank test was used to compare changes in group difference. The Cohen’s effect (ES) size was assessed for clinically relevant change in questionnaires measures (<0.3 low, 0.3-0.7 moderate, >0.7 strong difference).

## Results

The mean follow-up was 6.3 month for both groups. During the trial there were no adverse events. All surviving patients completed all surveys. Five patients (16.7%) in Arm A died within the first twelve weeks following RT, additional 7 patients (23.3%) died within 6 month due to tumor progression. In Arm B died 8 patients (26.7%) within 3 month and further 4 patients (13.3%) within 6 month. Mortality did not differ between groups.

Patients in the intervention group (Arm A) showed in psychosocial aspects a statistical significant advantage after three (p = 0.001) and six months (p = 0.010) (Table 2). The Cohen’s effect size confirmed the clinically significant improvement of the psychosocial aspects (ES -0.79).

**Table 2:** Effects of resistance training on Quality of life (EORTC QLQ-BM 22)

**Symptom scales**						
**Painful sites**	**Intervention group (Arm A)**			**Control group (Arm B)**		
	**n**	**Mean**	**SD**	**n**	**Mean**	**SD**
Baseline (t0)	30	39.78	23.91	30	39.56	24.51
3 month (t2)	25	29.6	19.73	22	35.76	27.1
6 month (t3)	18	22.22	13.14	18	35.93	32.67
Treatment effect (t0-t2) after 3 month p = 0.399, (t0-t3) after 6 month p = 0.445						
Effect size (t0-t2) after 3 month -0.24, (t0-t3) after 6 month -0.43						
**Pain characteristics**	**Intervention group (Arm A)**			**Control group (Arm B)**		
	**n**	**Mean**	**SD**	**n**	**Mean**	**SD**
Baseline (t0)	30	48.15	31.47	30	54.81	33.13
3 month (t2)	25	25.78	17.78	22	41.92	35.62
6 month (t3)	18	25.31	19.73	18	45.06	36.65
Treatment effect (t0-t2) after 3 month p = 0.905, (t0-t3) after 6 month p = 0.761						
Effect size (t0-t2) after 3 month -0.09, (t0-t3) after 6 month -0.24						
**Functional interference**	**Intervention group (Arm A)**			**Control group (Arm B)**		
	**n**	**Mean**	**SD**	**n**	**Mean**	**SD**
Baseline (t0)	30	55.14	26.32	30	55.28	28.95
3 month (t2)	25	35.33	20.35	22	44.7	30.38
6 month (t3)	18	29.86	20.77	18	48.38	30.12
Treatment effect (t0-t2) after 3 month p = 0.285, (t0-t3) after 6 month p = 0.081						
Effect size (t0-t2) after 3 month -0.21, (t0-t3) after 6 month -0.56						
**Psychosocial aspects**	**Intervention group (Arm A)**			**Control group (Arm B)**		
	**n**	**Mean**	**SD**	**n**	**Mean**	**SD**
Baseline (t0)	30	69.26	17.0	30	57.59	19.87
3 month (t2)	25	45.56	19.71	23	54.55	20.9
6 month (t3)	18	41.05	19.1	18	50.93	20.55
Treatment effect (t0-t2) after 3 month p = 0.001, (t0-t3) after 6 month p = 0.010						
Effect size (t0-t2) after 3 month -0.79, (t0-t3) after 6 month -0.77						

Treatment effect and Cohen’s effect size after 3 and 6 month.

The single item-analysis of psychosocial aspects showed in Arm A, worries regarding the loss of mobility were significantly lower after as early as three months (p = 0.007), and sustained after six months (p = 0.048), and were optimistic about their health. The control group, on the other hand, reported considerably more worries of becoming dependent on others (p = 0.015) (Table 3). Other scales of the EORTC QLQ-BM22 like painful site, and pain characteristics showed small to moderate ES without however becoming significant between groups in favoring to the intervention group. The functional interference showed a positive trend after six months (p = 0.081, ES -0.56).

**Table 3:** Item analysis of “psychosocial aspects” scale

	**Intervention group (Arm A)**						**Control group (Arm B)**						**Treatment effect**	
	**Baseline**		**3 month**		**6 month**		**Baseline**		**3 month**		**6 month**		**3 month**	**6 month**
**Item**	**Mean**	**SD**	**Mean**	**SD**	**Mean**	**SD**	**Mean**	**SD**	**Mean**	**SD**	**Mean**	**SD**	**p-value**	**p-value**
17	1.5	0.8	1.2	0.5	1.3	0.6	1.3	0.6	1.4	0.7	1.4	0.7	0.117	0.560
18	3.4	0.9	2.6	1.1	2.1	1.1	2.7	1.0	2.9	1.2	2.6	1.1	0.007	0.015
19	3.2	1.1	2.4	1.2	2.2	1.0	2.6	1.1	2.5	1.3	2.4	1.2	0.015	0.029
20	3.5	0.9	2.6	1.1	2.4	1.0	3.4	1.0	3.1	0.9	2.9	0.9	0.116	0.314
21	3.6	0.6	2.7	1.2	2.6	1.1	3.4	0.9	3.0	1.1	2.9	1.2	0.118	0.233
22	3.2	0.7	2.6	1.1	2.8	1.0	3.0	0.9	2.8	0.9	3.0	0.9	0.101	0.048
Questions														
17	Have you felt isolated from those close to you?													
18	Have you worried about loss of mobility because of your illness?													
19	Have you worried about becoming dependent on others because of your illness?													
20	Have you worried about your health in the future?													
21	Have you felt hopeful your pain will get better?													
22	Have you felt positive about your health?													

Results of the subscale (questions 17-22) according to EORTC QLQ-BM22.

After six months, patients in Arm A had significant lower physical fatigue (p = 0.013), and interference with daily life (p = 0.006) according to the FA13 assessment. Emotional and cognitive fatigue did not differ between groups, neither after 3 nor 6 months (Table 4). Finally, emotional distress was in the intervention group lower after six months (p = 0.016) (Table 5) compared to control group. The effect size confirmed these findings.

**Table 4:** Effects of resistance training on fatigue (EORTC QLQ-FA 13)

**Physical fatigue**	**Intervention group (Arm A)**			**Control group (Arm B)**		
	**n**	**Mean**	**SD**	**n**	**Mean**	**SD**
Baseline (t0)	30	57.22	29.0	30	58.06	29.1
After 3 month (t2)	25	49.0	24.92	22	56.44	30.53
After 6 month (t3)	18	35.65	25.37	18	64.91	31.25
Treatment effect (t0-t2) after 3 month p = 0.637, (t0-t3) after 6 month p = 0.013						
Effect size (t0-t2) after 3 month -0.04, (t0-t3) after 6 month -0.71						
**Emotional fatigue**	**Intervention group (Arm A)**			**Control group (Arm B)**		
	**n**	**Mean**	**SD**	**n**	**Mean**	**SD**
Baseline (t0)	30	46.67	32.5	30	44.44	30.27
After 3 month (t2)	25	33.67	25.63	22	43.18	34.85
After 6 month (t3)	18	27.31	27.54	18	46.05	33.26
Treatment effect (t0-t2) after 3 month p = 0.796, (t0-t3) after 6 month p = 0.156						
Effect size (t0-t2) after 3 month -0.14, (t0-t3) after 6 month -0.35						
**Cognitive fatigue**	**Intervention group (Arm A)**			**Control group (Arm B)**		
	**n**	**Mean**	**SD**	**n**	**Mean**	**SD**
Baseline (t0)	30	21.11	21.71	30	17.78	22.15
After 3 month (t2)	25	16.89	23.16	22	22.22	25.89
After 6 month (t3)	18	19.14	20.45	18	19.30	21.23
Treatment effect (t0-t2) after 3 month p = 0.248, (t0-t3) after 6 month p = 0.433						
Effect size (t0-t2) after 3 month -0.24, (t0-t3) after 6 month -0.19						
**Interference with daily life**	**Intervention group (Arm A)**			**Control group (Arm B)**		
	**n**	**Mean**	**SD**	**n**	**Mean**	**SD**
Baseline (t0)	30	2.53	0.94	30	2.30	1.09
After 3 month (t2)	25	2.20	1.04	22	2.64	1.00
After 6 month (t3)	18	1.89	0.96	18	3.05	0.97
Treatment effect (t0-t2) after 3 month p = 0.093, (t0-t3) after 6 month p = 0.006						
Effect size (t0-t2) after 3 month -0.48, (t0-t3) after 6 month -0.91						
**Social sequelae**	**Intervention group (Arm A)**			**Control group (Arm B)**		
	**n**	**Mean**	**SD**	**n**	**Mean**	**SD**
Baseline (t0)	30	1150	0.78	30	1.43	0.86
After 3 month (t2)	25	1.24	0.52	22	1.64	1.00
After 6 month (t3)	18	1.22	0.43	18	1.79	1.23
Treatment effect (t0-t2) after 3 month p = 0.129, (t0-t3) after 6 month p = 0.363						
Effect size (t0-t2) after 3 month -0.40, (t0-t3) after 6 month -0.37						

**Table 5:** Effects of emotional distress according to FBK-R10 questionnaire

**FBK-R10**	**Intervention group (n = 30)**			**Control group (n = 30)**		
	**n**	**Mean**	**SD**	**n**	**Mean**	**SD**
Baseline (t0)	30	24.7	10.8	30	24.13	12.1
3 month (t2)	25	18.84	9.2	22	22.41	11.8
6 month (t3)	18	15.44	8.6	18	24.95	12.8
Treatment effect (t0-t2) after 3 month p = 0.106, (t0-t3) after 6 month p = 0.016.						
Effect size (t0-t2) after 3 month -0.34, (t0-t3) after 6 month -0.87.						

## Discussion

Bone metastases frequently occur in advanced cancer diseases, and in the majority of cases are localized in the spinal column. The sequelae include pain, a raised risk of fracture, functional impairments and fatigue, all of them contributing to deteriorated in quality of life. After bone metastasis has been diagnosed, in many cases several weeks or even months pass with a considerable reduction in quality of life and persistent symptoms of pain. We therefore attempted to enhance patients’ QoL by combining resistance training to radiotherapy. To our knowledge, this randomized study is the first to investigate the benefit of isometric exercises to strengthen the paravertebral muscles of patients with spinal metastases on pain sensation, functional capacity, QoL and fatigue. Our results showed that patients report during and following radiotherapy a benefit from this adjunctive therapy compared to control group undergoing radiotherapy only, with regard to several domains of QoL and fatigue. Substantial improvement with small to moderate effect sizes could be observed in all dimensions of the EORTC QLQ-BM 22 (painful sites, pain characteristics, functional interference, and psychosocial aspects) compared to control group both after three and six months. However, with the exception of psychosocial aspects (in the EORTC QLQ-BM22 questionnaire) the difference between groups did not reach statistical significance; which is predominantly due to the small sample size. The psychosocial item was elicited in each individual question to identify at which points the difference was particularly apparent. In particular, patients in the intervention group reported worrying less about a potential loss of mobility as early as three months. After six months, the difference in worries became significant between groups. Patients who were regularly exercising also reported a more favourable state of health. By contrast, patients in the control group worried to a considerably greater extent on becoming dependent on others.

Some recent studies have provided indications of a large, clinically relevant short-term effect produced by exercise therapy [21]. Segal et al. reported that QoL assessed in FACT-General Scale was improved in a randomized study involving exercise training in patients with prostate cancer [22]. Ohira et al. showed a significant improvement in exercise training compared with a control group in the CARES-SF subscale for physical QoL and psychosocial QoL [23], indicating that exercise training is capable of exerting a positive effect on muscle growth and consequently on mobility in breast cancer survivors. In our study, patients in the intervention group were experiencing less pain-related “functional interference” and showed a positive trend after six months (p = 0.081).

As patients are experiencing increased performance status, this probably enhances the sense of control, independence, and self-esteem, which in turn leads to better social interactions and decreased anxiety and fear. To determine the clinical relevance by calculating Cohen’s effect size, a clinically significant improvement of the “psychosocial aspects’ scale” (ES -0.79) could be confirmed.

Cancer-related fatigue is defined as a persistent subjective sensation of tiredness relating to cancer treatment that impairs the patients’ physical and mental performance [24], and is the symptom of greatest clinical relevance for patients in an advanced stage of cancer disease [25]. Accumulating evidence from intervention studies indicates that exercise programmes are effective both in reducing the intensity of cancer-related fatigue and also help to prevent fatigue from becoming manifest [26-28].

In line with other authors’ findings, in our study resistance training was shown to result in substantial reduction of physical fatigue and interference with daily after 6 months whereas no changes in emotional and cognitive fatigue were observed.

Dimeo et al. [26] have already demonstrated that an exercise programme of limited duration not only improves the functional status and stamina, but also results in a major reduction in fatigue in cancer patients. Exercise therapy is a highly promising and effective therapeutic approach in sustainably combating cancer-related fatigue [20,22,29]. As demonstrated in this study, a simple-to-perform regimen of isometric exercises can be of substantial benefit, even to advanced-stage cancer patients. Our results showed a statistically significant benefit for the intervention group in all fatigue items, in particular regarding physical fatigue and interference with daily life. The intervention resulted in an improvement of mobility in daily life, which might have had an impact on reduced fatigue.

Moderate fatigue was shown in 66% of advanced stage cancer patients prior to radiotherapy [30]. This high prevalence of fatigue further underscores the importance of exercise therapy combined with palliative radiotherapy, respectively. Psychosocial distress, depression and anxiety are particularly prevalent in advanced disease stages, with proportions of clinical depression and anxiety patients ranging from 20 to 39% [31]. Our results showed a significant improvement in emotional distress and anxiety at 6-months follow-up, however, the effect size suggested a strong clinical effect. The effects of resistance training became apparent and significant in reduced sample after six months, what suggests only a benefit for survivors longer than six months. For planning further clinical trials in this setting, we recommend a Karnofsky performance score [14] < 70, and unstable metastases as exclusion criteria.

Because all patients were in advanced stages of their cancer, 40% of the patients in either group were lost to follow-up due to progressive disease and subsequently death. Further limitations of the study were the relatively small sample size, the variety of primary tumors and patient conditions, and the exclusion of patients presenting with cervical spine metastases. For feasibility reasons, patients’ compliance with the training program in their homes was assessed only by relying on patient-completed documentation forms.

Among the strengths of the study were the randomized design and a relatively low drop-out rate, as well as standardized and specific measures to assess multiple domains of QoL among patients with bone metastases. This is, to our knowledge, the very first application of a resistance exercise program in patients with spinal metastases concomitant to radiotherapy, to enhance their functional capacity and mobility, to reduce their fatigue and fears caused by spinal metastases, and thereby, to improve their QoL.

## Conclusion

In this group of patients we were able to show that guided isometric resistance training of the paravertebral muscles can improve functional capacity, reduce fatigue and thereby enhance QoL over a 6-months period in patients with stable spinal metastases. Importantly, the intervention was able to reduce specific fears around loss of mobility and depending on others‘assistance. This exercise is a promising and effective therapeutic approach to reduce emotional distress and anxiety specific to patients suffering from spinal metastases. Large controlled trials are necessary to confirm these findings.

## Competing interests

The authors declare that they have no competing interests.

## Authors’ contributions

HR and JD developed and planned this trial. TB is responsible for statistical considerations/basis of the analysis. GO, MA, and TW estimated the stability of bone metastases. HR, MK, SR, MH, and IS performed the examinations and RT supervisions. HR and AG made the data collection. HR performed the physical exercise. All authors read and approved the final manuscript.
